# The prevalence and characteristics of frailty in cirrhosis patients: a meta-analysis and systematic review

**DOI:** 10.3389/fmed.2024.1353406

**Published:** 2024-04-29

**Authors:** Ruiyu Xie, Xiaotong Jing, Chuanjie Yang

**Affiliations:** ^1^Department of Gastroenterology, The Second Hospital of Hebei Medical University, Hebei Key Laboratory of Gastroenterology, Hebei Institute of Gastroenterology, Hebei Clinical Research Center for Digestive Diseases, Shijiazhuang, Hebei, China; ^2^Department of Hematology, The Fourth Hospital of Hebei Medical University, Shijiazhuang, Hebei, China

**Keywords:** cirrhosis, frailty, prevalence, systematic review, meta-analysis

## Abstract

**Objectives:**

This study aimed to assess the prevalence of frailty in cirrhosis patients and the distribution of age, sex, and body mass index (BMI) in cirrhotic patients with frailty.

**Methods:**

We performed a thorough literature search using PubMed, Embase, Web of Science, and the Cochrane Library from inception to 29 February 2024. The estimated prevalence with a 95% confidence interval (CI) was calculated with a random effect model. Subgroup analysis and sensitivity analysis were performed to assess the heterogeneity and characterize the distribution of age, sex, and body mass index (BMI) in cirrhotic patients. Publication bias was assessed by the funnel plot, Begg's test, and Egger's test.

**Results:**

The 16 included studies, which were all observational, reported a prevalence of frailty in 8,406 cirrhosis patients ranging from 9 to 65%, and the overall estimated prevalence was 27% (95% CI: 21–33%; *I*^2^ = 97.7%, *P* < 0.001). This meta-analysis indicated that the estimated prevalence of frailty in cirrhosis patients was high, and compared to the non-frail cohort, the frail cohort tended to have a higher mean age, with a mean age of 63.3 (95% CI: 59.9, 66.7; *Z* = 36.48; *P* < 0.001), and a larger proportion of male patients with worse liver function, with a mean of 73.5% (95% CI: 71.4, 75.5%; *Z* = 7.65; *P* < 0.001), ND in the frail cohort, 54.8% (95% CI: 43.1, 66.5%; *P* < 0.001) and 23.4% (95% CI: 13.2, 33.7%; *P* < 0.001) were classified into Child-Pugh B and C, respectively. Meanwhile, the patients in the non-frail cohort are more likely to have a higher BMI, with a mean of 28.4 (95% CI: 24.1, 32.7; *Z* = 13.07; *P* < 0.001).

**Conclusion:**

The current study suggests that cirrhosis patients have a high prevalence of frailty. Compared with the non-frail cohort, the frail patients tend to be male, older, and have a lower BMI with worse liver function.

## Introduction

Frailty is a multidimensional clinical state of decreased physiologic reserve and increased vulnerability for patients. It is a condition in which all body systems gradually lose their capabilities, and it usually occurs in older people (1). However, as the definition of frailty evolves day by day in modern research, it has been observed in other diseases involving multiple systems, including end-stage liver diseases (2). The pathogenesis of frailty is complicated, and the possible theory describes the process as the combined influence of chronic inflammation, immune activation, and environmental and lifestyle factors (3). Currently, no agreement has been reached on the diagnosis of frailty, so various assessment instruments have been developed, such as the Edmonton Frailty Scale (EFS), Fatigue, Resistance, Ambulation, Illness, Loss of Weight (FRAIL) Index, and the Liver Frailty Index (LFI) (4, 5). The cost and prevalence of frailty are hard to evaluate due to the differences in the study population, sample size, and measurement instruments (6). Therefore, a synthesized analysis is needed to evaluate the frailty of certain diseases and to better prevent them.

Cirrhosis, on the other hand, is described as the final stage of chronic liver disease, combined with a series of complications (7). As the 11th most common cause of death (8), cirrhosis caused 1.7 million deaths worldwide in 2017, and the age-standardized death rate of cirrhosis is still rising (9). Frailty in cirrhosis patients has a great impact on mortality and life quality, especially for those awaiting transplants (10). Thus, considering the prevalence of cirrhosis and the impact of frailty, identifying the prevalence and characteristics of frailty in cirrhosis patients can be a lifesaver in end-stage liver disease management and, in the end, contribute to the primary, secondary, and tertiary prevention of liver disease.

Although previous studies have described the impact of frailty in cirrhosis patients, no unified conclusions have been reached on the estimated prevalence. Many factors, including mental health, unplanned hospital admissions, liver transplant waitlist mortality, age, and increased hospitalization days, are associated with frailty in cirrhosis patients. In addition, several high-quality observational studies that were published in recent years reported the prevalence of cirrhosis patients (11–26). Thus, we systematically gathered data from these articles to evaluate the prevalence and characteristics of frailty in cirrhosis patients.

## Methods

The study was conducted according to the Preferred Reporting Items for Systematic Reviews and Meta-Analysis guidelines (27). The protocol of this meta-analysis was registered by the Prospective Register of Systematic Reviews (PROSPERO) with the following registration number: CRD42023407442.

### Search strategy

We performed a thorough literature search using PubMed, Embase, Web of Science, and the Cochrane Library from inception to 29 February 2024. Key terms and the Medical Subject Headings (Mesh) terms were searched as follows: (“Liver Cirrhosis” OR “Hepatic Cirrhosis” OR “Liver Fibrosis”) AND (“Frailty” OR “Frail^*^” OR “Frailness” OR “Debilit^*^”). The comprehensive search process is presented in Supplementary Table S1. All matched articles, including systematic reviews and meta-analyses, were assessed during the search.

### Inclusion criteria

Studies involving the prevalence of frailty in patients with cirrhosis were included following these criteria: (1) the study is a cohort, cross-sectional, or any other observational, study; (2) patients were diagnosed with cirrhosis by medical records or clinical findings; (3) frailty was diagnosed by a standardized and validated index, such as the Liver Frailty Index (LFI) and Carolina Frailty Index (CFI), or clinical evaluations; and (4) the study population is adult (over 18 years old).

### Exclusion criteria

Articles will be excluded after a comprehensive examination if they meet the following criteria: (1) the article is a study protocol, case report, conference abstract, or any other type of article that is not original; (2) the article is a duplicate; and (3) the article has irrelevant outcomes.

### Study selection

The selection was performed independently by two reviewers (RX and XJ) by checking titles and abstracts to exclude irrelevant studies. The full text of selected articles will be assessed to determine whether they are eligible. A senior reviewer (MW) carried out the final assessment when there was a disagreement between authors performing the screening.

### Data extraction

Following the guideline for data extraction for systematic reviews and meta-analysis, two reviewers (RX and XJ) independently worked on eligible articles, collecting the following information: author, country, year of publication, study design, the diagnosis of frailty, age, sex distribution, body mass index (BMI), and the number of cirrhosis patients with or without frailty. A discussion will be held to settle any disagreements with a third reviewer (MW).

### Risk of bias assessment

To assess the quality of articles included in our meta-analysis, a modified tool (28) consisting of 10 items covering four domains of bias was used during the process. The total score of the individual observational study was from 0 to 10, and every single item was valued at 0 or 1. The study was classified into low, moderate, and high quality with a total score of 0–5, 6–8, and 9–10.

### Statistical analysis

Considering the characteristics of frailty events and total cirrhotic patients, we used a random effect model with the double-arcsine transformation to perform the meta-analysis to better calculate the estimated prevalence of frailty in patients with liver fibrosis. The chi-squared test and *I*^2^ value were calculated to assess heterogeneity. If the *P*-value is < 0.1 or *I*^2^ is > 50%, then the heterogeneity would be considered high, and we would conduct a random effect model for pool analysis. Furthermore, subgroup analysis would be performed to characterize the distribution of age, sex, and BMI in such patients. The funnel plot, Egger's test, and Begg's test were combined to assess the publication bias both visually and statistically. All data in our study were analyzed by Stata/MP 14.0, and a *P*-value of < 0.05 was considered significant in statistical analysis.

## Results

### Database search and study selection

**Figure 1:**
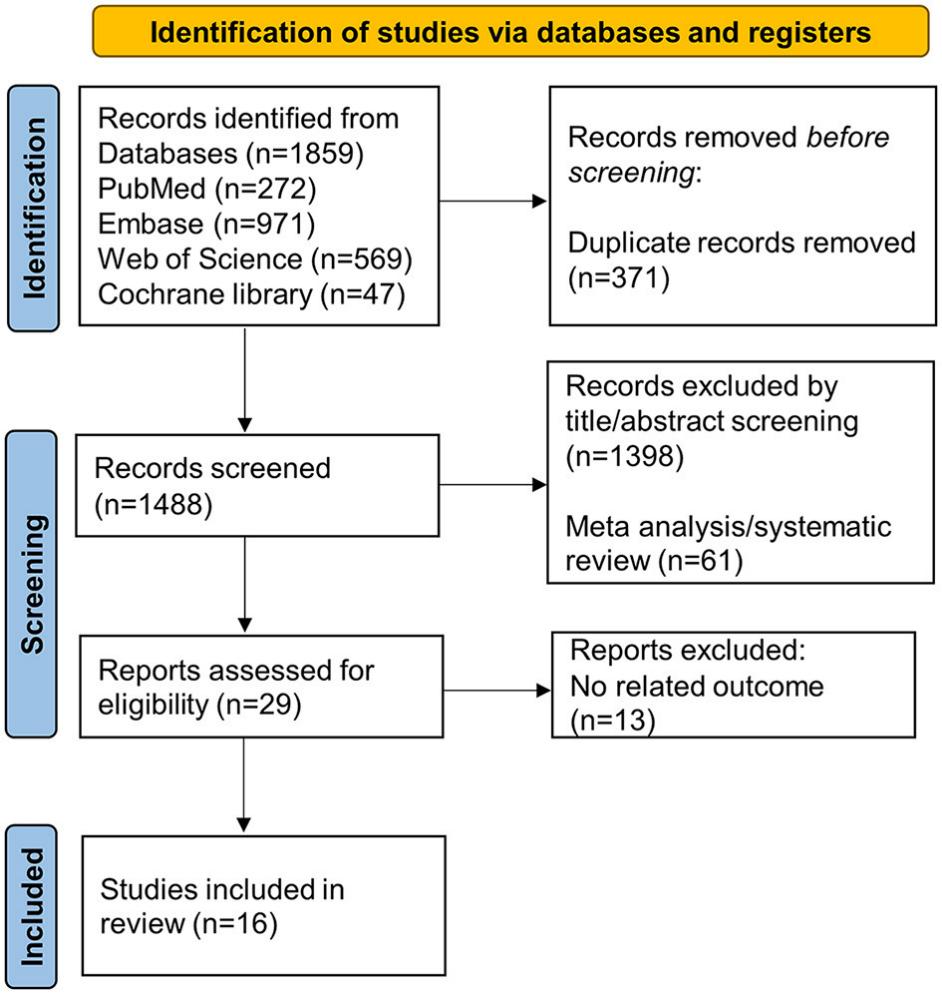
The process of study selection.

At the end of our search, 29 February 2024, a total of 1,859 studies were retrieved from the databases; among them, 371 were duplicated, and 1,488 records, including 61 meta-analyses, systematic reviews, and review articles, were excluded after viewing titles and abstracts. After assessing the full articles of the remaining 29 studies, 16 studies were considered eligible for our meta-analysis. Figure 1 shows the PRISMA flowchart describing study selection and screening.

### Study characteristics

In conclusion, in 16 observational studies (11–26), 8,406 cirrhosis patients were included, with sample sizes ranging from 126 to 1,623. The eligible studies, with data gathered from China, Chile, Japan, Slovakia, Spain, Thailand, and the United States, were published from 2018 to 2023. The baseline characteristics of the included articles are shown in Table 1.

**Table 1:** Baseline characteristics of the study population.

**References**	**Year**	**Country**	**Study design**	**Diagnosis**	**Age**	**sample size**	**M/F**	**BMI**	**Frail**	**Non-frail**
Siyu et al. (23)	2023	China	Cross-sectional study	The Liver Frailty Index	(–)	387	(–)	(–)	39	348
Mao et al. (17)	2023	China	Retrospective cohort study	Carolina Frailty Index with minor modifications	63 (57, 69)	245	111/134	24.3 (21.3, 27.3)	27	218
Cullaro et al. (13)	2022	United States	Prospective cohort study	The Liver Frailty Index	58 (50, 63)	1,033	589/444	(–)	313	720
Berry et al. (12)	2022	United States	Cohort study	The Liver Frailty Index	58 (50, 63)	1,623	949/674	28.3 (24.9, 32.6)	451	1,172
Bajaj et al. (11)	2022	United States	Prospective cohort study	The clinical frailty scale	(–)	442	(–)	(–)	40	402
Xu et al. (26)	2021	United States	Prospective cohort study	The Liver Frailty Index	(–)	1,623	(–)	(–)	451	1,172
Soto et al. (25)	2021	Chile	Prospective cohort study	Clinical evaluation	64 ± 8.3	126	62/60	29.4 ± 4.8	82	44
Skladany et al. (24)	2021	Slovakia	Cohort study	The Liver Frailty Index	(–)	385	291/94	(–)	184	201
Siramolpiwat et al. (22)	2021	Thailand	Cohort study	The Liver Frailty Index	62.5 ± 9.3	152	87/65	(–)	37	115
Serper et al. (21)	2021	United States	Prospective cohort study	The Liver Frailty Index	57 ± 12	211	115/96	30.0 ± 7.0	124	87
Roman et al. (19)	2021	Spain	Prospective cohort study	Five Fried Frailty criteria of the cardiovascular health study	(–)	135	97/38	(–)	35	100
Feng et al. (15)	2021	China	Cohort study	Carolina Frailty Index	63 (55, 68)	202	98/104	23.7 (20.5, 26.5)	35	167
Deng et al. (14)	2021	United States	Cohort study	The Liver Frailty Index	61 (54–65)	233	134/99	29 (25–33)	43	190
Saeki et al. (20)	2020	Japan	Cross-sectional study	Fried's five components	70 (59–76)	291	137/154	23.1 (20.8–26.0)	81	210
Lai et al. (16)	2020	United States	Cohort study	The Liver Frailty Index	60 (53–64)	983	649/334	28 (25–32)	151	832
Puchades et al. (18)	2018	Spain	Cross-sectional study	The Liver Frailty Index	60 (53–65)	335	221/114	28 (25–33)	53	282

### Quality assessment

There were 13 cohort studies and three cross-sectional studies included in our study, and the average score of them was 8.92 and 9.00, respectively, indicating the eligible studies had high quality. Among all the included studies, 11 cohort studies (13–17, 19, 21, 22, 24–26) and two cross-sectional studies (18, 20) were rated over 9, which were classified as high-quality articles, and the remaining articles were deemed moderate quality. The detailed score is shown in Table 2.

**Table 2:** Risk of bias in the included articles.

**Study items**	**Publication year**	**1**	**2**	**3**	**4**	**5**	**6**	**7**	**8**	**9**	**10**	**Scores**	**Overall quality**
**Cohort studies**													
Mao et al. (17)	2023	1	1	1	0	1	1	1	1	1	1	9	High
Cullaro et al. (13)	2022	1	1	1	1	1	1	1	1	0	1	9	High
Berry et al. (12)	2022	1	1	1	0	1	1	1	1	0	1	8	Moderate
Bajaj et al. (11)	2022	0	1	0	1	1	1	1	1	1	1	8	Moderate
Xu et al. (26)	2021	1	1	1	0	1	1	1	1	1	1	9	High
Soto et al. (25)	2021	1	1	1	0	1	1	1	1	1	1	9	High
Skladany et al. (24)	2021	1	1	1	1	1	1	1	0	1	1	9	High
Siramolpiwat et al. (22)	2021	1	1	1	0	1	1	1	1	1	1	9	High
Serper et al. (21)	2021	1	1	1	1	1	0	1	1	1	1	9	High
Roman et al. (19)	2021	1	1	1	1	1	1	1	1	1	1	10	High
Feng et al. (15)	2021	1	1	1	0	1	1	1	1	1	1	9	High
Lai et al. (2)	2021	1	1	1	1	1	0	1	1	1	1	9	High
Deng et al. (14)	2020	1	0	1	1	1	1	1	1	1	1	9	High
**Cross-sectional studies**													
Siyu et al. (23)	2023	1	1	1	1	1	0	1	1	0	1	8	Moderate
Saeki et al. (20)	2020	1	1	1	1	1	1	0	1	1	1	9	High
Puchades et al. (18)	2018	1	1	1	1	1	1	1	1	1	1	10	High

1. Was the study's target population a close representation of the national population about relevant variables? 2. Was the sampling frame a true or close representation of the target population? 3. Was some form of random selection used to select the sample, or was a census undertaken? 4. Was the likelihood of nonresponse bias minimal? 5. Were data collected directly from the subjects (as opposed to a proxy)? 6. Was an acceptable case definition used in the study? 7. Was the study instrument that measured the parameter of interest shown to have validity and reliability? 8. Was the same mode of data collection used for all subjects? 9. Was the length of the shortest prevalence period for the parameter of interest appropriate? 10. Were the numerator(s) and denominator(s) for the parameter of interest appropriate?

### The prevalence of frailty in cirrhosis patients

**Figure 2:**
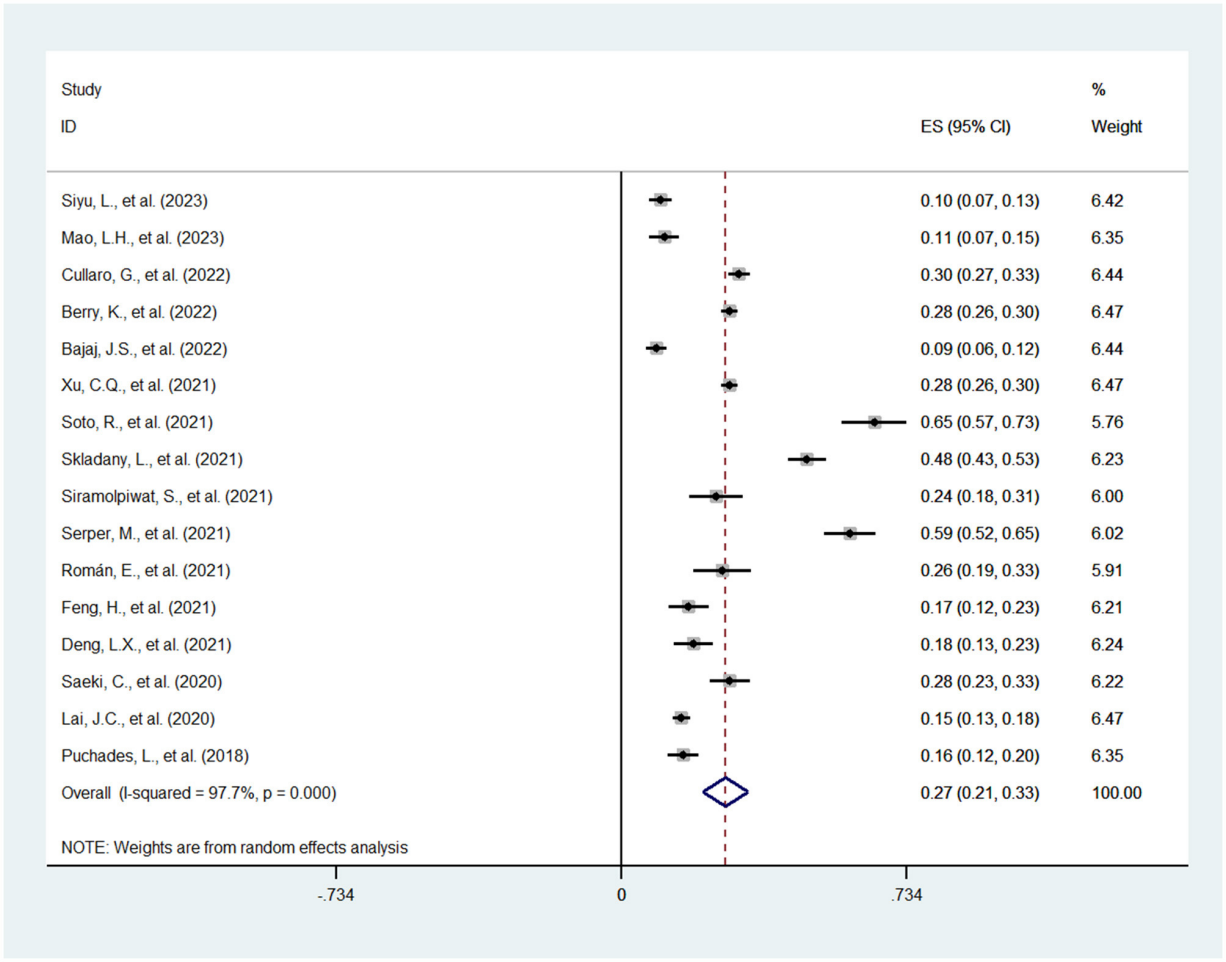
Meta-analysis of the prevalence of frailty in cirrhosis patients.

The 16 included studies (11–26), which were all observational, reported a prevalence of frailty in cirrhosis patients ranging from 9 to 65%, and the overall estimated prevalence was 27% (95% CI: 21–33%; *I*^2^ = 97.7%, *P* < 0.001). The detailed result is displayed in Figure 2. Through the process, the heterogeneity of the data was examined by sensitivity analysis to find the possible cause, and none of the individual studies reversed the pooled-effect size, as shown in Supplementary Figure S1, which suggested the high stability of our study.

### Subgroup analysis

To better characterize the distribution of age, sex, and BMI in cirrhotic patients with frailty, we performed meta-analyses for subgroups separately in the frail and non-frail cohorts from five studies (19, 21–23, 25). Through the subgroup analysis, we found that the cirrhosis patients in the frail cohort tend to have a higher age, with a mean age of 63.3 (95% CI: 59.9, 66.7; *Z* = 36.48; *P* < 0.001), and a larger proportion of male patients, with a mean of 73.5% (95% CI: 71.4, 75.5%; *Z* = 7.65; *P* < 0.001). Meanwhile, the patients in the non-frail cohort are more likely to have a high BMI, with a mean of 28.4 (95% CI: 24.1, 32.7; *Z* = 13.07; *P* < 0.001). The detailed result is displayed in Table 3. Additionally, the researchers conducted meta-analyses on the frail and non-frail cohorts to discuss the distribution of the Child-Pugh class in cirrhosis patients, as shown in Table 4. Compared to the frail population, up to 53.6% (95% CI: 29.0, 78.2%; *P* < 0.001) of non-frail patients were classified into Child-Pugh A, with a lower proportion of the patients classified into Child-Pugh B and Child-Pugh C at 39.2% (95% CI: 20.3, 58.2%; *P* < 0.001) and 12.3% (95% CI: 8.8, 15.7%; *P* < 0.001), respectively.

**Table 3:** Subgroup analysis of the distribution of age, sex, and BMI in frail and non-frail cirrhosis patients.

	**Frail**	**Non-frail**
Age (years)	63.3 (95% CI: 59.9, 66.7; *Z* = 36.48; *P* < 0.001)	59.5 (95% CI: 54.7, 64.3; *Z* = 24.38; *P* < 0.001)
BMI	28.1 (95% CI: 26.0, 30.1; *Z* = 26.95; *P* < 0.001)	28.4 (95% CI: 24.1, 32.7; *Z* = 13.07; *P* < 0.001)
Male (%)	73.5% (95% CI: 71.4, 75.5%; *Z* = 7.65; *P* < 0.001)	54.7% (95% CI: 52.2, 57.1%; *Z* = 20.52, *P* < 0.001)

**Table 4:** Subgroup analysis of the proportion of different Child-Pugh classes in frail and non-frail cirrhosis patients.

	**Frail**	**Non-frail**
A	30.0% (95% CI: 10.7, 49.3%; *P* < 0.001)	53.6% (95% CI: 29.0, 78.2%; *P* < 0.001)
B	54.8% (95% CI: 43.1, 66.5%; *P* < 0.001)	39.2% (95% CI: 20.3, 58.2%; *P* < 0.001)
C	23.4% (95% CI: 13.2, 33.7%; *P* < 0.001)	12.3% (95% CI: 8.8, 15.7%; *P* < 0.001)

### Publication bias

**Figure 3:**
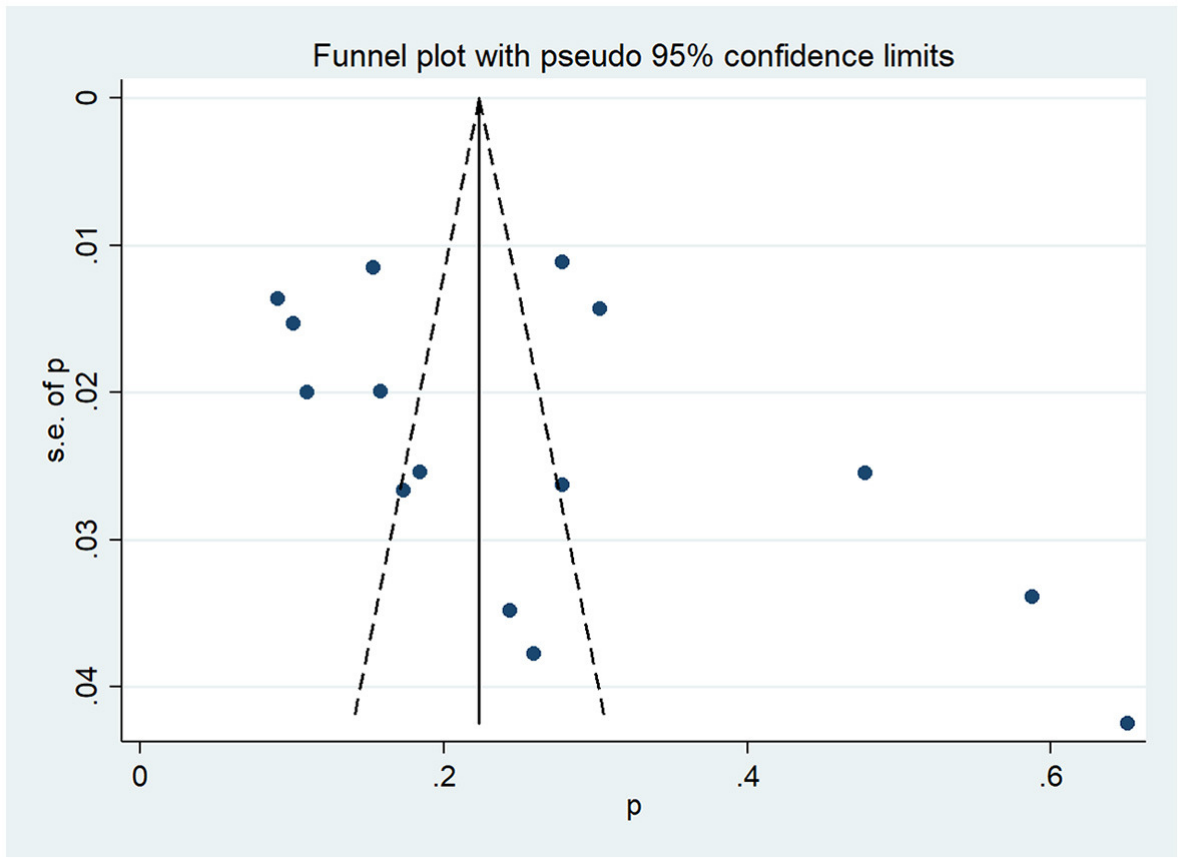
The funnel plot of the prevalence of frailty in cirrhosis patients.

To examine whether there was a publication bias, we conducted Begg's test and Egger's test, which resulted in PBegg = 0.150 (*P* > 0.05) and PEgger = 0.200 (*P* > 0.05), indicating that no publication bias was observed in our study statistically. Visually, the symmetrical funnel plot is shown in Figure 3, which also proves the same conclusion.

## Discussion

Our meta-analysis compared the outcomes of data collected from 16 observational studies regarding the prevalence of frailty in 8,406 patients with cirrhosis. An estimated prevalence of 27% was shown in cirrhosis patients with frailty. We further investigated the distribution of sex, age, and BMI in cirrhosis patients with or without frailty to characterize our target patients. As a result, the frail cohort has a higher average age, a larger proportion of male patients, and a lower BMI than the non-frail cohort. Such results can help clinicians to easily and swiftly identify frailty in patients with cirrhosis.

At the time our research is being conducted, few research studies have focused on the prevalence of frailty in cirrhosis patients. A previous meta-analysis (29) discussed the frailty assessment instruments in cirrhosis patients, including the Liver Frailty Index (LFI), the Short Physical Performance Battery, the 5-m gait speed, and routine nursing assessment, but it did not research the estimated prevalence of frailty as our study does. Currently, the most frequent tool is the LFI, which is a performance-based tool comprising three separate tests, including grip strength, chair stands, and balance testing. The other most commonly used tools are the Fried phenotype and the Fried Frailty Index (FFI), which cover weight loss, exhaustion, low physical activity, slowness, and weakness. Other tools include the Karnofsky Performance Scale (KPS), which assesses patients' ability to work and care for themselves, and the short physical performance battery (SPPB), which includes a balance test, gait speed test, and chair stand test. Several other meta-analyses (30–34) calculated the prevalence of frailty in different populations, which resulted in 11% in the older community-dwelling population, 53% in long-term care residents, 5%−29% in patients with human immunodeficiency virus (HIV) infections, and 37% in patients with end-stage renal disease. All the studies mentioned above discussed a specific population without cirrhosis. Overall, our study filled the gap in the prevalence of frailty in cirrhosis patients.

There have been several reports demonstrating the association of factors with frailty in cirrhosis patients, including mental health, unplanned hospital admissions, liver transplant waitlist mortality, age, and increased hospitalization days. For instance, researchers have identified age as a significant influencing factor for frailty (35). Our findings also reveal that the cirrhosis patients in the frail cohort tend to have a higher age when compared to the non-frail cohort. However, it should be noted that a recent report found that cirrhosis patients may also experience frailty at a younger age (23). Together with our findings, aged cirrhosis patients require more frequent evaluation in clinics.

In the current study, a male predominance of frailty among cirrhosis was found, which is against the published findings (19, 25). First, these studies and a few others included a lot more male than female patients, which may create bias. Second, male patients with cirrhosis are more likely to have comorbidities such as spontaneous bacterial peritonitis and hepatocellular carcinoma, which may also accelerate frailty.

With our findings, we hope cirrhosis patients can be identified swiftly and easily during outpatient visits and inpatient admissions to improve the quality of life and mortality in end-stage liver disease patients. Considering the prevalence of frailty in cirrhosis patients, all kinds of assessment instruments should be used regarding local demographics in hepatology clinical practice. After the diagnosis of frailty, a comprehensive intervention combining in-hospital treatment with community-based physical activity and nutritional programs (36) should be taken to reduce the prevalence and improve mortality in end-stage liver disease patients, especially those on the liver transplant waitlist.

To better summarize our meta-analysis, we are focusing on the prevalence of frailty in cirrhosis patients and calculating related parameters through subgroup analysis. The result of the sensitivity analysis and assessing the risk of bias in prevalence studies through the modified tool (28) indicated the credibility and stability of our study. There are also limitations in our meta-analysis. First, the study population in articles meeting the inclusion criteria comes from a diverse background, including age, nationality, race, etc., which can cause bias. Second, during the research, high heterogeneity was found. As limited data was retrieved, the cause of heterogeneity was unable to be identified. Third, we failed to acquire sufficient data regarding the complications, etiology, and other factors that might be influencing the prevalence of frailty in cirrhosis patients. Fourth, the data on the clinical impact of frailty, such as the severity of liver cirrhosis, in the reported papers chosen is limited. Future work could explore the clinical impact for the benefit of clinical practice. Finally, funnel plot asymmetry cannot discriminate between publication bias and other sources of asymmetry, and meta-regression could be employed to assess the heterogeneity.

## Conclusion

This meta-analysis indicated that the estimated prevalence of frailty in cirrhosis patients stayed at a high level, and compared to the non-frail cohort, the frail patients tend to be male, older, and have a lower BMI with worse liver function. With these findings, we hope more resources and efforts can be directed toward reducing the prevalence of frailty in cirrhosis patients and improving their mortality. This approach could potentially lead to better health outcomes and quality of life for these patients.

## Data availability statement

The original contributions presented in the study are included in the article/Supplementary material, further inquiries can be directed to the corresponding author.

## Author contributions

RX: Data curation, Validation, Writing – original draft, Writing – review & editing. XJ: Data curation, Validation, Writing – original draft, Writing – review & editing. CY: Data curation, Funding acquisition, Resources, Supervision, Validation, Writing – original draft, Writing – review & editing.
