# Supplementary material for: The prevalence and characteristics of frailty in cirrhosis patients: a meta-analysis and systematic review
Source: Front Med (Lausanne). 2024 Apr 29;11:1353406. doi: 10.3389/fmed.2024.1353406 (PMC11092890; doi:10.3389/fmed.2024.1353406)
Supplement: Supplementary file 2 [file Data_Sheet_2.DOCX]

**Supplementary materials**


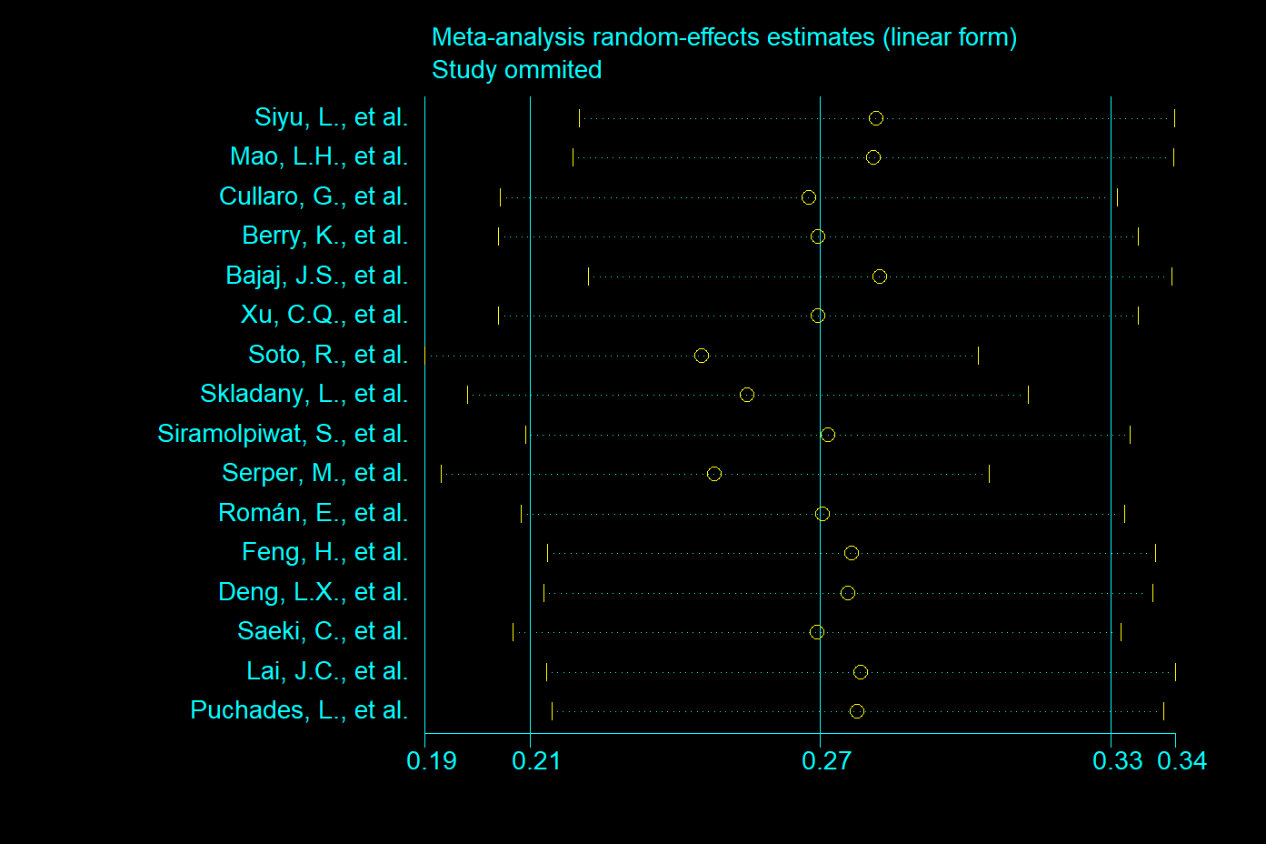


Figure S1. Meta-analysis random-effects estimates.

Table S1. Details of the Literature Search Strategy.

(1) PubMed (February 29, 2024)

| Search | Query | Results |
| --- | --- | --- |
| 1 | "Frailty"[Mesh] | 10379 |
| 2 | ("Frailty"[Mesh]) OR (((Frailt*[Title/Abstract]) OR (Frailness[Title/Abstract])) OR (Debilit*[Title/Abstract])) | 63535 |
| 3 | "Liver Cirrhosis"[Mesh] | 104515 |
| 4 | ("Liver Cirrhosis"[Mesh]) OR ((Hepatic Cirrhosis[Title/Abstract]) OR (Liver Fibrosis[Title/Abstract])) | 114686 |
| 5 | (("Liver Cirrhosis"[Mesh]) OR ((Hepatic Cirrhosis[Title/Abstract]) OR (Liver Fibrosis[Title/Abstract]))) AND (("Frailty"[Mesh]) OR (((Frailt*[Title/Abstract]) OR (Frailness[Title/Abstract])) OR (Debilit*[Title/Abstract]))) | 272 |

(2) Cochrane Library (February 29, 2024)

| Search | Query | Results |
| --- | --- | --- |
| 1 | MeSH descriptor: [Liver Cirrhosis] explode all trees | 8245 |
| 2 | ("Hepatic Cirrhosis"):ti,ab,kw OR ("Liver Fibrosis"):ti,ab,kw (Word variations have been searched) | 2043 |
| 3 | #1 OR #2 | 9748 |
| 4 | MeSH descriptor: [Frailty] explode all trees | 722 |
| 5 | (Frailt*):ti,ab,kw OR (Frailness):ti,ab,kw OR (Debilit*):ti,ab,kw (Word variations have been searched) | 7280 |
| 6 | #4 OR #5 | 7280 |
| 7 | #3 AND #6 | 47 |

(3) Embase (February 29, 2024)

| Search | Query | Results |
| --- | --- | --- |
| 1 | 'frailty'/exp | 29578 |
| 2 | frailt*:ab,ti OR frailness:ab,ti OR debilit*:ab,ti | 91708 |
| 3 | #1 OR #2 | 96448 |
| 4 | 'liver cirrhosis'/exp | 212493 |
| 5 | 'hepatic cirrhosis':ab,ti OR 'liver fibrosis':ab,ti | 43340 |
| 6 | #4 OR #5 | 240259 |
| 7 | #3 AND #6 | 971 |

(4) Web of science (February 29, 2024)

| search | query | results |
| --- | --- | --- |
| 1 | ((TS=(Liver Cirrhosis)) OR TS=(Hepatic Cirrhosis)) OR TS=(Liver Fibrosis) | 145812 |
| 2 | (((TS=(Frailty)) OR TS=(Frailt*)) OR TS=(Frailness)) OR TS=(Debilit*) | 70946 |
| 3 | #2 AND #1 | 569 |
